# Molecular speciation and transformation of soil legacy phosphorus with and without long-term phosphorus fertilization: Insights from bulk and microprobe spectroscopy

**DOI:** 10.1038/s41598-017-13498-7

**Published:** 2017-11-10

**Authors:** Jin Liu, Jianjun Yang, Barbara J. Cade-Menun, Yongfeng Hu, Jumei Li, Chang Peng, Yibing Ma

**Affiliations:** 1 Institute of Agricultural Resources and Regional Planning, Chinese Academy of Agricultural Sciences, Beijing, 100081 China; 2 Institute of Environment and Sustainable Development in Agriculture, Chinese Academy of Agricultural Sciences, Beijing, 100081 China; 3 Agriculture and Agri-Food Canada, Swift Current Research and Development Centre, Box 1030, Swift Current, SK S9H 3X2 Canada; 4 Canadian Light Source, University of Saskatchewan, Saskatoon, SK S7N 2V3 Canada; 5 Agriculture Environment and Resources Center, Jilin Academy of Agricultural Sciences, Jilin, 130033 China

## Abstract

Soil legacy phosphorus (P) represents a substantial secondary P resource to postpone the global P crisis. To fully utilize this P reserve, the transformation of legacy P speciation in a black soil with and without P fertilization for 27 years was investigated by chemical fractionation, molecular-level bulk (P K-edge X-ray absorption near-edge, XANES; solution ^31^P nuclear magnetic resonance) and microprobe (µ-X-ray fluorescence and µ-XANES) spectroscopy. Results from both fractionation and P bulk-XANES concordantly indicated that Ca_2_-P [Ca(H_2_PO_4_)_2_] acts as a reserve of labile P in response to soils with or without P fertilization. Cropping for 27 years depleted hydroxyapatite while enriched iron-bound P in soils irrespective of P application. Similar accumulation of soil organic P (P_o_), probably due to root residue inputs, occurred in both soils with and without P fertilization; the accumulated P_o_ was present as orthophosphate diesters in soils with P fertilization more than in soils without P fertilization, suggesting that the release of labile P_o_ was triggered by soil P deficits. These results provide vital information for agronomically and environmentally sustainable P management by demonstrating the potential crop availability of legacy soil P, which could reduce future P fertilization.

## Introduction

Phosphorus (P) is a non-substitutable nutrient for agricultural production; however, the world reserve of rock phosphate used to produce fertilizers is finite and is being rapidly depleted^1^. Over the past decades, the large accumulation of surplus P in cropland soils due to the excessive and intensive application of P fertilizers, termed soil legacy P^2–4^, has threatened water quality and agricultural profitability^5–7^. Over the period 1965–2007, cumulative P application (550 kg ha^−1^) was more than twice the crop cumulative uptake (225 kg ha^−1^) globally^8^. Although there are spatial variations^8,9^, global stocks of soil legacy P are considerable^3^. So far, soil legacy P represents a substantial secondary P resource to postpone the worldwide P crisis^4^. It has been estimated that soil legacy P could support global crop P demands for approximately 9–22 years depending on its availability^4,10^. Therefore, more sustainable P management that efficiently exploits soil legacy P for crop production has become a global concern, with agronomic, environmental and economic benefits^4,11^.

The routine approach for P management is based on soil test methods (e.g. Olsen P, Colwell P), which neglect the contribution of soil legacy P to crop P uptake^4,12^. Consequently, in many cases, crop yield does not show a reduction with lower soil test P when decreasing or even halting P application^13,14^. This suggests that P uptake is being replenished by legacy P that cannot be measured by current soil test methods^15^. Soil legacy P exists in various chemical species with a continuum of availability (generally classified as readily available, sparingly available, and very stable P)^16^. All forms of P exist in complex equilibria through abiotic (precipitation-dissolution and adsorption-desorption between the solid phase and soil solution) and biotic (immobilization-mineralization between inorganic and organic forms) processes, which differ with parent material, soil pH, land use, and pedogenesis^16–19^. As such, rational P management should consider the replenishing ability of soil legacy P under specific environmental conditions^9,15^, particularly the extent to which and for how long current legacy P could support crop uptake, as well as when and how much new P fertilizer should be added to prevent yield reduction^4^. This was highlighted by studies on the patterns of build-up and drawdown of soil legacy P and its plant-availability, which depends on the forms that are accumulated and depleted with and without P fertilization^13,20^. Although multiple strategies for soil, crop and nutrient management need to be integrated^4,12^, understanding the nature and dynamics of legacy P in croplands is still the key priority to fully utilize soil legacy P.

Soil P chemistry and/or transformation have been investigated with various soil types through P fractionation methods^21,22^. Sequential fractionation is relatively simple and feasible, yet suffers from fastidious extraction steps and the poor resolution of specific P composition^23,24^. Fortunately, recent advances in analytical chemistry, especially synchrotron-based X-ray absorption near-edge structure (XANES) and solution nuclear magnetic resonance (NMR) spectroscopies, allow the speciation of P at the molecular level^25–27^. With P K-edge XANES, different inorganic P (P_i_) species (e.g., Ca-, Fe-, and Al-bound P) with unique spectral features can be distinguished in a non-disruptive way through fingerprinting analysis, and their quantities can be estimated with linear combination fitting (LCF)^28–30^. Solution ^31^P nuclear magnetic resonance (NMR) spectroscopy provides information on the relative abundance of specific organic P (P_o_) forms and classes (e.g., phosphonates, orthophosphate monoesters, and diesters)^31–33^. However, the relatively low concentration and highly heterogeneous chemical bonding environments of P in soils can decrease the specificity while increasing the uncertainty of soil P characterization by these advanced approaches for many soils^30,34^. For example, P K-edge XANES provides the average information on P speciation, and is not sensitive enough to discriminate more than five chemical species of P in most soil samples^34^. To overcome these limitations, spatially resolved spectroscopies, e.g. micro X-ray fluorescence (µ-XRF) and microscopically focused XANES (µ-XANES), particularly in the tender X-ray range, have been developed over the last few years^35^. At the microscale, µ-XRF provides *in situ* spatial information about P and its bonding elements in diverse matrices, while µ-XANES allows for probing P in micron-scale spots, which may contain fewer P species, or have P distributed heterogeneously^36–38^. Despite the shortcomings of individual techniques, the best approach for the most comprehensive understanding of a complex system is, undoubtedly, to combine complementary techniques that allow confirmation of a given observation^39^.

China plays a key role in global sustainable P management^3,40^. From 1970 to 2010, the total P surplus through chemical P application in Chinese croplands was 56 Tg, which was more than twice the global chemical P production in 2010^40^. Black soils (Mollisols in USDA Soil Taxonomy)^41^, which are rich in organic carbon (C) and P and are typically fertile and productive, play a vital role in crop production in China^42^. An ongoing long-term field experiment that was established in 1989 provides one of the few resources to study aspects of agricultural sustainability for black soils in China that cannot be reliably based on short-term trials, including soil fertility, nutrient cycling, and fertilizer-yield response^43,44^. Two treatments of this long-term experiment were chosen in this study, one with mineral P plus nitrogen (N) and potassium (K) fertilization (NPK), and the other with only N and K without P fertilization (NK). We hypothesized that 27 years of continuous cropping with and without P fertilization would affect the speciation, abundance, and transformation of soil legacy P. Combining state-of-the-art spectroscopic (bulk/µ-XANES, µ-XRF and solution P-NMR) analyses with fractionation, the objective of this study was to understand the forms of soil legacy P that accumulate or contribute to crop nutrition with or without P fertilization.

## Results and Discussion

### Changes in soil properties and crop yields with and without P fertilization

The 27 years of NK and NPK fertilization induced no significant variations in soil total C and total N concentrations (Table S1). From 1989 to 2015, soil total P dropped by 10% (to 440.5 mg kg^−1^) without P fertilization, while increasing by 34% (to 654.6 mg kg^−1^) with P application (Fig. S2 and Table S1). This suggests that soil legacy P was retained or depleted over time with or without P fertilization, respectively. A similar trend also occurred for Olsen-P, which reflected the different regimes of P management. In contrast, the 2015 soils had higher soil P_o_ concentrations than the 1989 soil, regardless of P fertilization, although the percentage of P_o_ increased only in the NK treatment (Table S1). Furthermore, soil pH significantly decreased with and without P fertilization over the 27 years, probably due to the long-term urea N fertilization^45^. Generally, the three-year moving averages of maize yields increased with P fertilization and decreased without P fertilization over the 27 years, relative to the starting year (Fig. S1). Assuming legacy P would not limit crop uptake until the maize yield decreased by more than 10% (that is yield less than 7558 kg ha^−1^), it could be estimated, using the regression equation of Fig. S1, that legacy P could support crop production for approximately 20 years.

### Changes in soil P pools with and without P fertilization

**Figure 1:**
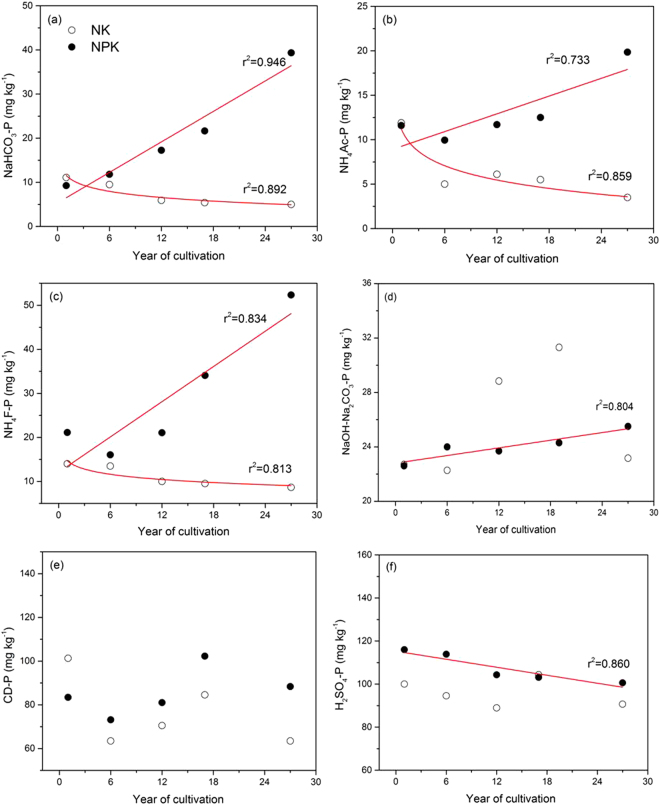
Phosphorus sequential fractionation for the studied soils with (NPK, solid circles) and without P application (NK, open circles) sampled at intervals from 5 to 10 years for the 27-year long-term experiment. Fitted curves for NPK treatment: *y* = a*x* + b, while those for NK treatment: *y* = a*lnx* + b. CD-P represented citrate dithionite extracted phosphorus. Year 1 is 1989, and year 27 is 2015.

Using sequential fractionation, the long-term changes in soil P pool distribution from 1989–2015 were studied using archived soil samples under NK and NPK treatments at intervals from 5 to 10 years (Fig. 1). On average, the fractionation method extracted 90% of soil total P_i_ (range 72–111%). Regardless of the treatment and duration, the dominant P fractions in these soils were NaOH-Na_2_CO_3_-extractable (Fe-P), H_2_SO_4_-extractable (Ca_10_-P) and citrate dithionite (CD)-extractable P (occluded P), which collectively accounted for 65–92% of the total extracted P. Less than 35% of P was extracted with NaHCO_3_ (Ca_2_-P and labile sorbed P), NH_4_Ac (Ca_8_-P), and NH_4_F (Al-P). Studies on black soils using a similar extraction method also showed the predominance of occluded P^46,47^.

Over 27 years of cultivation, the Ca_2_-P and labile sorbed P pool declined from 11.1 mg kg^−1^ to 5.0 mg kg^−1^ (55%) under the NK treatment, but increased from 9.3 mg kg^−1^ to 39.4 mg kg^−1^ (323%) under the NPK treatment (Fig. 1a). This suggests that this P pool was depleted by maize uptake without P fertilization but was greatly enriched with P application. A similar pattern was also reported with respect to changes in Olsen-P (Table S1), which is comparable to the Ca_2_-P and labile sorbed P fraction^14,48^. Additionally, 27 years of cropping without P application reduced the Ca_8_-P (from 11.9 mg kg^−1^ to 5.0 mg kg^−1^, Fig. 1b) and Al-P fractions (from 14.0 mg kg^−1^ to 8.7 mg kg^−1^, Fig. 1c). Based on regression analysis, decreases in these P fractions under the NK treatment could be described by a logarithm function of time (R^2^ > 0.813, Fig. 1a–c), but not the changes in the other P fractions. In contrast, these fractions (Ca_8_-P, Al-P) and Fe-P accumulated in the NPK treatment with soil P buildup, with all of these P fractions exhibiting linear increases over time (R^2^ > 0.733, Fig. 1b–d). This suggested that Ca_8_-P and Al-P act as reserves of potentially labile P for maize uptake when soil legacy P is depleted. Soil P buildup in moderately labile P fractions with fertilization has previously been reported^15,49^, although other studies report that these P pools remained stable or declined^50^. Furthermore, linear decline occurred for the Ca_10_-P pool extracted with H_2_SO_4_ (R^2^ = 0.860, Fig. 1f) under the NPK treatment. Given that sequential fractionation is a destructive and operationally-defined method, advanced spectroscopic techniques, as employed in the following part of this study, are vital to reveal more precise insights into the specific P forms and their changes among treatments^22,24^.

### Changes in soil inorganic P with and without P fertilization

**Figure 2:**
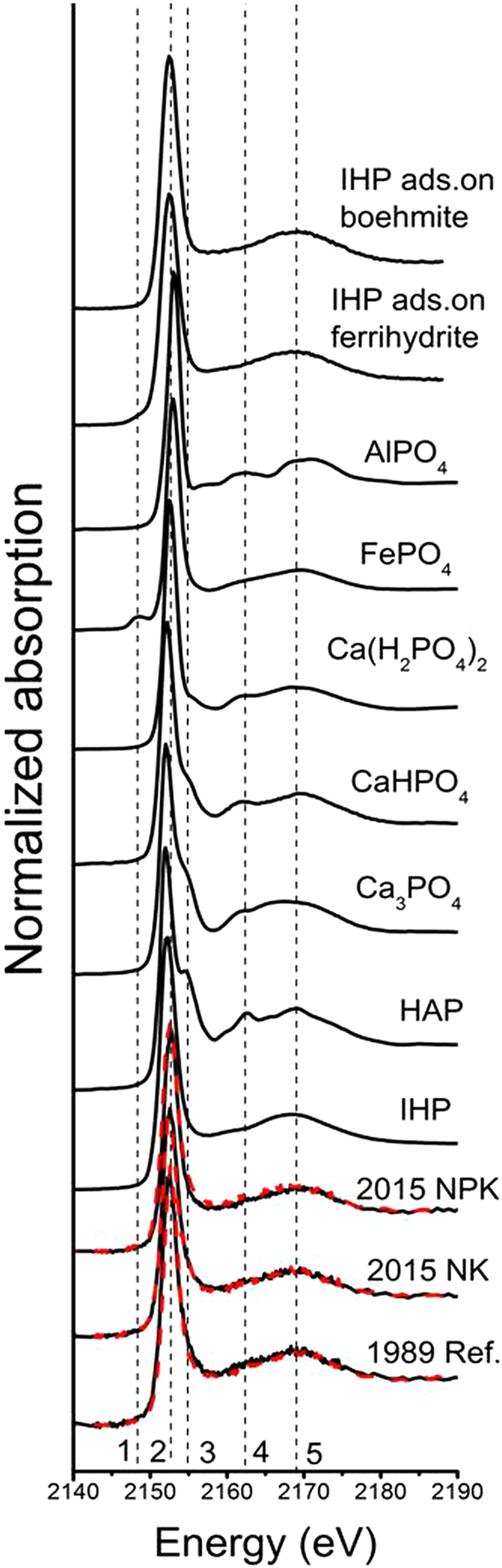
Bulk P K-edge XANES spectra of the selected P standards and soil samples including the reference soil collected in 1989 (Ref.) as a baseline and those collected in 2015 from the long-term plots with (NPK) and without (NK) 27 years of P fertilization. The red dashed lines represent the lineal combination fittings of the P K-edge XANES spectra of the studied soil samples. Identified P compounds in soils are shown in Table 1. The vertical dashed lines indicate spectral features for different P species: peak 1, Fe- P; peaks 2, main peak; peak 3 and 4, Ca-P; peak 5, oxygen oscillation. IHP, inositol hexakisphosphate, IHP ads. on boehmite and ferrihydrite, IHP adsorbed on boehmite and ferrihydrite, respectively. HAP, hydroxyapatite.

Synchrotron-based P K-edge bulk-XANES and microprobe (µ-XRF and µ-XANES) spectroscopies were applied to characterize the molecular speciation of P_i_ in the soils sampled in 1989 before the experiment establishment (reference soil) and in 2015. The P K-edge bulk-XANES spectra of the investigated P standards showed distinguishing spectral features in the pre- and post-edge regions (Fig. 2), as are commonly reported^28,30,37^. In brief, all Ca-bound P standards exhibited features on the post-edge (peaks 3 and 4), and the intensity of peak 3 depicted a decreasing order of hydroxyapatite (HAP) > Ca_3_(PO_4_)_2_ > CaHPO_4_ > Ca(H_2_PO_4_)_2_. The pre-edge feature (peak 1) was observed in the spectra of Fe-bound P, including FePO_4_ and inositol hexakisphosphates (IHP) adsorbed on ferrihydrite. Aluminum-bound P (AlPO_4_) showed a post-edge feature (peak 4) similar to Ca-bound P, but lacked the pre-edge feature of Fe-bound P. The spectrum of IHP was the most featureless and had a broad white line peak (peak 2). Thus, these distinct fingerprints of XANES spectra allow for the identification of P forms represented by these standards and a reasonable quantitative analysis thereafter.

**Figure 3:**
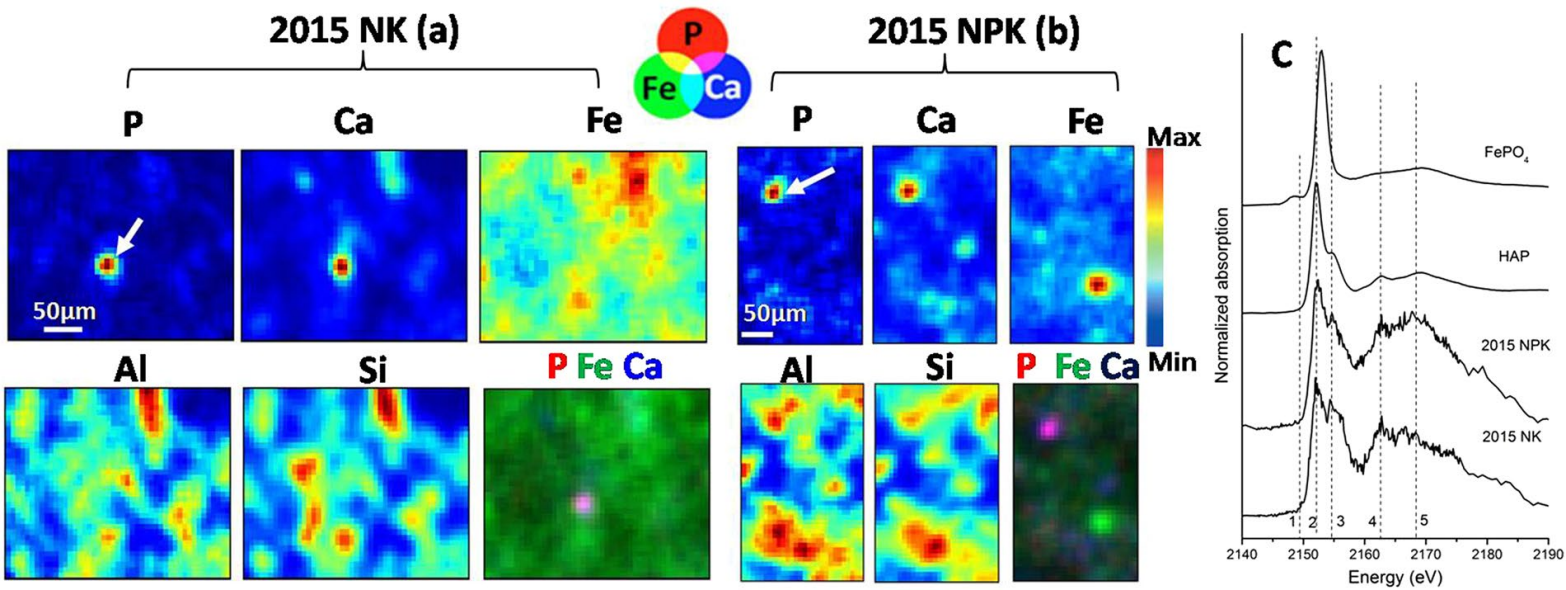
Distribution of P, Fe, Al, Ca, and Si in soils sampled in 2015 from the long-term plots without (NK, **a**) and with (NPK, **b**) 27 years of P fertilization by μ-XRF mapping and P speciation at the selected hop spots (where indicated with the white arrows) probed by μ-XANES (**c**). Individual signals of each element are shown with concentration proportional to brightness and RGB composite map of P, Fe and Ca are also exhibited. Spectral peaks of interest are marked by vertical dashed lines and labeled from1 to 5: peak 1, Fe- P; peak 2, main peak; peak 3 and 4, Ca-P; peak 5, oxygen oscillation.

By visual inspection, P K-edge XANES of the soil samples resembled the standard spectrum of IHP (Fig. 2), which suggested the presence of organically-bound P in the investigated soils. Additionally, the spectral broadening on the high-energy side of the white line (peak 3) suggested that a portion of P could be Ca-bound. This was more clearly demonstrated by the spatially resolved spectroscopy (Fig. 3), which provided complementary information to bulk-XANES analysis. In this study, µ-XRF maps for the 2015-NK and NPK soils displayed the presence of minute P hot spots disseminated in a low-concentration diffuse background, in line with the results of Rivard and co-authors^37^. The overlapping discrete hot spots of P and Ca agree well with the good correlation of P with Ca (R^2^ > 0.578) but the poor correlation with other elements in the 2015 soils (Fe, Al and Si; R^2^ < 0.215; Fig. S3). Further µ-XANES spectra were collected at specific points of interest on these soils. These spectra, with features on peak 3 and 4, matched well with the bulk-XANES spectrum of HAP (Fig. 3c). Therefore, the micron-scale observations presented in this study visually confirmed the dominance of HAP at P hot spots of the 2015 soils. Additionally, the pre-edge peak 1 in µ-XANES spectra of the 2015 soils indicated the presence of Fe-P, particularly for the NK soil (Fig. 3c), which did not appear in the bulk-XANES spectra (Fig. 2). The presence of Fe-P seems reasonable given the wide distribution of Fe (hydr)oxides as P sorbents in the 2015 soils as indicated by the diffusion of Fe signals by µ-XRF (Fig. 3a and b). Bulk-XANES provides the averaged structural information on the local binding of P with soil constituents. However, generally, soil P hot spots are extremely localized as indicated in our results and those of Rivard *et al*.^37^. Thus, the true nature of P species, particularly those present in low abundance or with fewer peak features, probably could be obscured or even concealed by the low-concentration diffuse background when performing P bulk-XANES analysis. So far, micro-analysis at P K-edge for soils is relatively rare, but is becoming more available^35,38^. As such, we urge more researchers to use µ-XRF and µ-XANES spectroscopies wherever possible to compare with observations by bulk-XANES analysis for a reliable and comprehensive P speciation.

Linear combination fitting, as a more quantitative alternative to visual inspection and fingerprinting comparisons, was conducted to differentiate the P species represented among treatments (Fig. 2 and Table 1)^51^. According to the LCF results of the normalized bulk-XANES spectra, Ca(H_2_PO_4_)_2_ accumulated in the 2015 NPK soil compared to the 1989 reference soil but was undetected in the 2015 NK soil (Table 1). This supports the trend of sequential fractionation results showing the enrichment and depletion of the labile Ca_2_-P pool in soils with and without P fertilization, respectively (Fig. 1). These observations imply that Ca_2_-P acts as a labile reserve of soil legacy P in response to soil P fertilization or depletion. Additionally, relative to the 1989 reference soil, a noticeable decrease in HAP and an increase in FePO_4_ occurred in both 2015 soils regardless of P fertilization (Table 1), which was partially supported by the decreased Ca_10_-P and increased Fe-P fractions under NPK treatment (Fig. 1d and f). These results suggest the potential release of HAP from the soil legacy P pool for maize uptake or transformation to other P species (e.g., Fe-P) in these black soils. Relative to the reference soil, soil acidification under both 2015 treatments (~1.8 pH unit, Table S1) could be an important driving force for HAP depletion, given HAP dissolution increases with decreasing soil pH^16^. Plant roots could induce the release of insoluble soil Ca-P through rhizospheric pH decreases and ligand complexation^29,52,53^. The results presented here are important, demonstrating long-term field-based evidence of soil HAP depletion. Once labile orthophosphate is released from HAP into the soil solution, it may associate with Fe (hydr)oxides to form Fe-P species, given the high affinity of orthophosphate to Fe (hydr)oxides, and may be subsequently stored as an intermediate reserve in the soil. This probably accounted for the increase of FePO_4_ in the 2015 soils (Table 1). The enrichment of FePO_4_ in the NPK treatment, supported by our fractionation results (Fig. 1d), agrees well with the observations that P fertilization contributed to the Fe-P pool^15,49,50^. Future studies are warranted to determine whether the Fe-P, determined as FePO_4_ based on our XANES analysis, is present as less bioavailable occluded-P or more bioavailable sorbed P on Fe oxyhydroxides in these soils with contrasting P fertilization.

**Table 1:** Phosphorus species^a^ in the studied soil samples by bulk P K-edge XANES fitting.

Treatment^b^	Linear combination fitting				Goodness of fit	
	Ca(H_2_PO_4_)_2_	FePO_4_	IHP	HAP	R factor	**χ** ^**2**^
	*Proportion* (*%*)					
1989-Reference	20 ± 2	21 ± 1	31 ± 3	28 ± 2	0.0013	0.0037
2015-NK	0	39 ± 1	49 ± 3	12 ± 2	0.0019	0.0059
2015-NPK	34 ± 2	35 ± 1	21 ± 2	10 ± 1	0.0008	0.0020
	*Concentration* (*mg kg* ^−1^)					
1989-Reference	98 ± 10	103 ± 5	152 ± 15	137 ± 10		
2015-NK	0	172 ± 4	216 ± 13	53 ± 9		
2015-NPK	223 ± 13	229 ± 6	137 ± 13	65 ± 6		
	*Changes of P concentrations in treatment relative to 1989 reference* (*%*)					
Without P fertilizer^c^	−100	+67	+42	−61		
With P fertilizer^c^	+127	+123	−9	−52		

^a^IHP, inositol hexakisphosphate; HAP, hydroxyapatite. ^b^1989-Reference, the soil sampled before the establishment of the experiment as a reference, 2015-NPK and NK, the soil sampled in 2015 from the long-term experiment with and without 27-year P fertilization, respectively. ^c^The ratio of differences in concentrations of each P species between the 2015 samples with and without P and the 1989 reference soil, expressed in percentage.

This study, for the first time, directly probed soil legacy P_i_ in black soils at the molecular level by multiple advanced techniques including synchrotron-based P K-edge bulk-XANES, µ-XRF and µ-XANES, rather than operationally-defined P pools by fractionation in previous publications^46,54^. The good quality of LCF fits (R factors < 0.0019, Table 1) in this study indicated that species in the investigated samples were matched well with the chosen standards. Given the inherent limitations of P K-edge XANES analysis such as the uncertainty in P standard selection and subsequent LCF routines for compound identification^34,55^, the data presented here were interpreted with caution, and quantitative results on specific soil P species might not be perfectly accurate. Nevertheless, the changes among treatments revealed in our study were supported by multiple lines of evidence, which made the key results of this study reliable and reasonable.

### Changes in soil organic P with and without P fertilization

**Figure 4:**
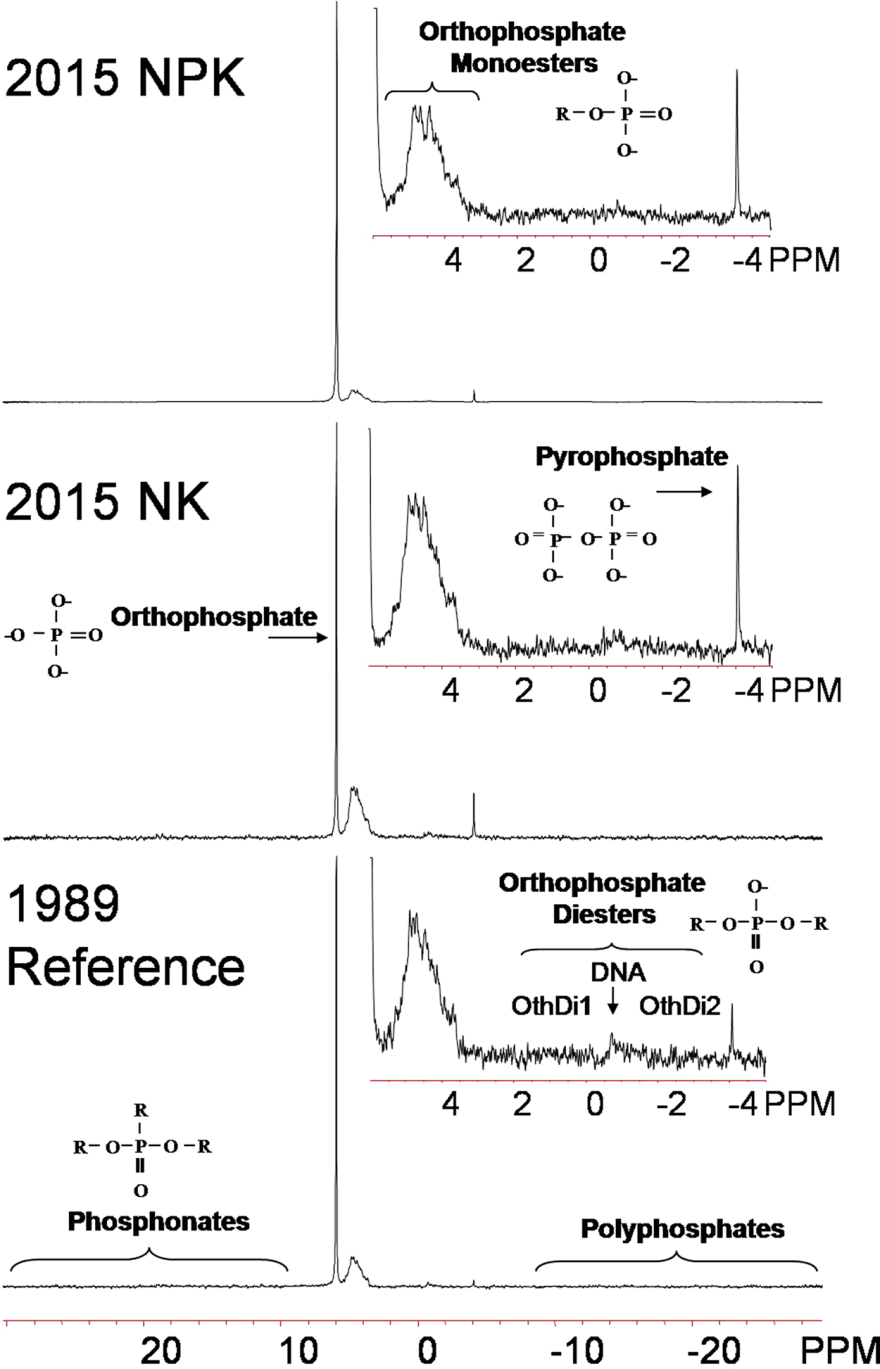
Phosphorus NMR spectra of soil samples including the reference soil collected in 1989 and those collected in 2015 from long-term plots with (NPK) and without (NK) P fertilization for 27 years. The main spectrum is plotted in full with 7 Hz line-broadening; insets showing the orthophosphate monoester and diesters regions are plotted with 3 Hz line-broadening.

Solution P-NMR spectroscopy was applied to speciate soil P_o_ in the aforementioned black soils, including 1989 reference, 2015-NK and 2015-NPK soils. Among treatments, the P extraction efficiency of NaOH-EDTA ranged from 33 to 49% of total P (Table 2), which agrees with other P-NMR studies on soils^32^. Compare to the XANES analysis, solution P-NMR is not as sensitive to the speciation of P_i_ forms such as orthophosphate. However, solution P-NMR characterized biogenic P forms: (1) complex P_i_, including polyphosphate and pyrophosphate; (2) phosphonates; (3) orthophosphate monoesters, including stereoisomers of inositol hexakisphosphate (*myo-*, *scyllo-*, *neo-*, and D-*chiro*-IHP), choline phosphate, glucose 6-phosphate, glucose 1-phosphate, and degradation products of orthophosphate diesters (α- and β-glycerophosphate, mononucleotides); and (4) orthophosphate diesters, which were mainly DNA in these soils (Figs 4, S4 and Table S2). Other unidentified orthophosphate monoesters and diesters were grouped into general categories (Mono1, Mono2, Mono3, Di1, Di2; Figs 4, S4 and Table S2). Because mononucleotides, α-, and β-glycerophosphate resulted from the degradation of compounds that were orthophosphate diesters in the original soil during NMR extraction and analysis^33,56^, the percentages of monoesters and diesters were also calculated another way by including these compounds within the diesters rather than the monoesters (Table 2).

**Table 2:** Phosphorus form classes or ratios of form classes^a^ in the NaOH-EDTA extractions determined by integration of P-NMR signals.

Treatment^b^	NaOH-EDTA extraction									
	Extracted total P	P_i_	P_o_	total IHP	Mono	Di	D:M	Cmono	Cdi	cD:M
*Proportion* (*%*)										
1989-Reference	33^c^	44.7	55.3	19.9	45.3	5.4	0.12	36.4	14.3	0.39
2015-NK	40^c^	34.8	65.2	24.6	56.9	5.3	0.09	46.0	16.2	0.35
2015-NPK	49^c^	65.5	34.5	12.6	27.4	4.0	0.15	21.1	10.3	0.49
*Concentration* (*mg kg* ^−1^)										
1989-Reference	163	72.9	90.1	32.4	73.8	8.8	—	59.3	23.3	—
2015-NK	176	61.2	114.8	43.3	100.1	9.3	—	81.0	28.5	—
2015-NPK	323	211.6	111.4	40.7	88.5	12.9	—	68.2	33.3	—
*Changes of P concentrations in treatments relative to 1989 reference* (*%*)										
Without P fertilizer^d^	—	−16	+27	+34	+36	+6	−25	+36	+22	−10
With P fertilizer^d^	—	+190	+24	+26	+20	+47	+25	+15	+43	+26

^a^P_i_, inorganic P; P_o_, organic P; total IHP, the total of inositol hexakisphosphate; Mono, orthophosphate monoester; Di, orthophosphate diesters; D:M, the ratio of orthophosphate diesters to orthophosphate monoester; C denotes a correction for degradation products. ^b^1989-Reference, the soil sampled before the establishment of the experiment as a reference, 2015-NPK and NK, the soil sampled in 2015 from the long-term experiment with and without 27-year P fertilization, respectively. ^c^The ratio of NaOH-EDTA extracted P to soil total P. ^d^The ratio of differences in concentrations of each P species between the 2015 samples with and without P and the 1989 reference soil, expressed in percentage.

Organic P represented 34.5–65.2% of the NaOH-EDTA extracted P in the studied soils by NMR (Table 2). Proportions of P_o_ forms decreased in the order of orthophosphate monoesters > orthophosphate diesters > phosphonates (3.0–4.6%, Tables 2 and S1). Before correction for degradation, orthophosphate monoesters comprised 27.4 to 56.9% of extracted P, which dropped to 21.1 to 46.0% after correction. The proportion of diesters rose from 4.0 to 5.4% to 10.3 to 16.2% after correction (Table 2). For all treatments, the most dominant orthophosphate monoester group was IHP, representing 12.6–24.6% of NaOH-EDTA extracted P (Table 2). Of the IHP stereoisomers, *myo*-IHP was the most abundant (33.3–41.2% of total IHP, Table S2). The second largest P_o_ compound class was monoester2 (5.7–15.8%, Table S2), which is a general category of P forms that may contain sugar phosphates, lower inositol phosphates, and other degradation products of orthophosphate diesters if present^33,57^. Within the orthophosphate diester range, DNA was the only specifically identified compound and accounted for the largest proportion (40.0–57.4% of diesters, Table S2). Inorganic polyphosphate (<2.3%) and pyrophosphate (<3.8%, Table S2), both originating from microbial activity^58^, only accounted for trace percentages.

In our study, it is interesting and particularly important to note that similar increases occurred for concentrations of P_o_ in both 2015 soils (114.8 and 111.4 mg kg^−1^) with 27 years of cropping history but contrasting P fertilizer management, relative to the 1989 reference soil (90.1 mg kg^−1^, Table 2). A similar trend was observed for the concentration of total soil P_o_ by the ignition method (Table S1). Without manure and biosolids application, soil P_o_ predominantly originates from crop inputs (roots and residues) and microbial immobilization^59^. While the microbial biomass P only represents a minor proportion (0.7–2.5%) of total P in croplands^60^, all crops and straw were removed from the plots at harvest in this long-term experiment, and thus residual maize roots were probably the major source of P_o_ accumulation in the soils over time. Previous studies indicated the presence of orthophosphate monoesters (i.e. choline phosphate, glucose-6-phosphate, etc.) and diester compounds (i.e. DNA) in maize roots^61,62^. The contribution of maize root residues to P_o_ concentrations in these black soils over 27 years of cultivation was further supported by the increases in both orthophosphate monoesters and diesters in the 2015 soils, regardless of P fertilization, relative to the 1989 reference soil (Table 2). Before and after correction for degradation, NK treatment enriched the concentration of soil orthophosphate monoesters (by 36% without and with correction for degradation) to a greater extent than the NPK treatment (by 20% and 15%), and the concentrations of diesters to a lesser extent (by 6% and 22% for NK vs. by 47% and 43% for NPK, Table 2). The similarities in the accumulation of total P_o_ by maize root residues but differences in distribution of the increased P_o_ between orthophosphate monoesters and diesters suggest the preferential depletion of orthophosphate diesters and secondary enrichment of monoesters when P status was low under NK treatment. Orthophosphate diesters generally represent the readily labile P_o_ pool, degrading to orthophosphate and orthophosphate monoesters. Biologically mediated mobilization and mineralization of otherwise unavailable P_o_, through microbial proliferation and symbiotic associations with mycorrhizal fungi, could be triggered by P deficiency when soil C and N concentrations are sufficient for microbial needs^63,64^. Even IHP, generally considered to be very stable orthophosphate monoester, can be mobilized to phytoavailable P with adequate available C and N sources^65^. The ratio of orthophosphate diesters to monoesters (D:M), an indicator of P degradation^26^, varied among treatments, which further supported the above hypothesis. As compared to the 1989 reference soil, the uncorrected (D:M) and corrected (cD:M) ratios decreased in the 2015 NK soil (Table 2), indicating a net depletion of diester compounds without P fertilization. In contrast, higher cD:M and D:M ratios in the 2015 NPK soil (Table 2) revealed a net accumulation of diesters. Meanwhile, IHP, as the major orthophosphate monoester constituent, accumulated during P depletion under NK treatment, which was consistent with the bulk P K-edge XANES results (Table 1). Similar enrichment of IHP appeared under NPK treatment by NMR data, although only a small change was shown by P K-edge XANES analysis. This discrepancy could be explained by the insensitivity of P K-edge XANES for P_o_ speciation, which has been reported elsewhere^27,29^.

Consistent with the hypothesis described above, changes in the concentrations of individual biogenic P species reflected differences in P biochemistry among the treatments. Specifically, cultivation reduced the enrichment of labile diesters (OthDi1, and OthDi2) under NK treatment (8%, and 9%) relative to the NPK treatment (111%, and 98%, Table S2). In contrast, few changes in DNA concentrations were observed among treatments (Table S2). This is not surprising because DNA tends to be protected from dephosphorylation by adsorption to the soil matrix, while other diesters (e.g. phospholipids and RNA, grouped as the OthDi1 and OthDi2 in this study) are vulnerable to biodegradation and therefore would be preferentially biodegraded for crop uptake under soil P deficits^66^. Like DNA, *myo*-IHP remains stable due to soil stabilization^66^, and showed little change among treatments (Table S2). The higher increase of *scyllo*-IHP, D-*chiro*-IHP (4e/2a), choline phosphate, glucose 1-phosphate, glucose 6-phosphate, and Mono 2 in the 2015 NK than the NPK treatment may be linked to the bio-formation of these P_o_ species in response to soil P depletion. The relatively lower increases in concentrations of *neo*-IHP, phosphonates, pyrophosphate, Mono1, and β-glycerophosphate might result from a depletion of these P compounds to generate orthophosphate. Due to the incomplete extraction of P by NaOH-EDTA and the risk of hydrolysis during analysis, the quantitative NMR results on specific soil P species might not be perfectly accurate and should be interpreted with caution, similar to the XANES data. In the current study, the depletion of soil labile P_o_ species under NK treatment and enrichment under NPK treatment implies the utilization of soil legacy P_o_ in the black soils to supply crop needs. This suggests that fertilizer inputs could be reduced to drawdown legacy P reserves without compromising crop yields, as was recently reported for grassland soils in Northern Ireland^20^. This in turn could reduce P loss to water from legacy P^5^. However, further research is needed for individual soils and crops.

## Conclusions

Our study provided clear evidence that without P fertilization for 27 years, soil legacy P_i_ was depleted for crop uptake over time, mainly as Ca(H_2_PO_4_)_2_ and HAP. Soil legacy P_o_ transformations among treatments suggested that the release of orthophosphate from labile orthophosphate diester could be triggered by soil P deficits. Phosphorus fertilization enriched labile P_i_ forms and some P_o_ forms such as orthophosphate diesters, probably derived from maize root residues. As such, the replenishment of soil legacy P to readily available orthophosphate must be comprehensively considered to optimize fertilizer management, and this requires a full understanding of soil P speciation and dynamics. Collectively, these results will be vital to provide a theoretical basis to reduce P fertilization, thereby realizing the potential of soil legacy P, at least in P-enriched black soils, to improve agricultural sustainability while mitigating environmental deterioration. Further research is warranted to estimate the quantity of soil legacy P actually available to crops. Additionally, the exact processes and mechanisms regulating the release of soil legacy P under given environmental conditions remain elusive and deserve further study.

## Methods

### Soil sampling

The long-term fertilization experiment under a continuous maize monoculture system, designed to investigate black soil fertility and fertilizer effects, was initiated in 1989 at Gongzhuling, Jilin province, China (43°30′N, 124°48′E). The soil is classified as a Mollisol in USDA Soil Taxonomy^41^, and has been cropped with P addition for at least 100 years^44^. Prior to the establishment of the experiment, maize was cropped without fertilizer application for 3 years (1987–1989) to make the soils more homogeneous (i.e. variance of maize yields among plots less than 10%)^42^. For the present study, two fertilization treatments were selected from the plots receiving annual applications of nitrogen (urea, 165 kg N ha^−1^) and potassium (KCl, 68.5 kg K ha^−1^) with P (superphosphate) application rates of 0 (NK) and 36 kg P ha^−1^ (NPK) in a randomized complete block design with three replicates. More details of the experimental site and climate conditions are provided elsewhere^42,44,48^.

Before the experiment establishment, five cores of soils (0–20 cm) were taken and mixed into one as the 1989 reference soil. Five fresh soil samples (0–20 cm) were collected towards the center of each plot after maize harvest annually, and then composited. Soil samples were mixed thoroughly, air-dried, sieved, and stored for analysis. Soil pH was measured in deionized water (1:2.5 w/v). Total P was digested with H_2_SO_4_-HClO_4_^67^, total P_o_ was determined by the ignition method^68^, and Olsen-P was determined with sodium bicarbonate extraction^69^, all followed by colorimetric analysis^70^. Total C and total N were determined by an element analyzer (VarioMax). Selected properties of the soil sampled before the establishment of the experiment in 1989 and those collected in 2015 are given in Table S1.

### Phosphorus fractionation

Archived soil samples with two treatments collected in 1989, 1994, 2000, 2007, and 2015 were fractionated using the method proposed by Jiang and Gu^71^, and subsequently modified by Adhami *et al*.^72^. This method was introduced based on methods by Chang and Jackson^73^ and Hedley *et al*.^74^, both of which are more widely used. However, we chose this method for this study because it is especially suitable for calcareous soils, and more importantly, it is a more chemically precise method compared to some other fractionation methods^71,75^. Soil P was successively extracted with NaHCO_3_, NH_4_Ac, MgCl_2_, NH_4_F, NaOH-Na_2_CO_3_, CD, and H_2_SO_4_. The first step (NaHCO_3_) is thought to remove Ca_2_-P and labile sorbed P^76^, and NH_4_Ac targets moderately labile Ca_8_-P. The third extraction with MgCl_2_ is to prevent re-adsorption of P onto CaCO_3_. Then NH_4_F separates Al-P from Fe-P, while NaOH-Na_2_CO_3_ extracts Fe-P. Occluded P is removed in the CD step, H_2_SO_4_ removes Ca_10_-P. Samples are washed twice with 95% alcohol after the first step and with saturated NaCl after the other steps to remove any residual P left from each previous step. Inorganic P in each extract was analyzed colorimetrically using the molybdate blue method^70^. Magnesium chloride-extracted P was grouped in the NH_4_Ac fraction. The interference of CD and NH_4_F on P determination was eliminated by digesting with HClO_4_-H_2_SO_4_-HNO_3_ (1:2:7) mixtures and adding boric acid, respectively^71,72^. To remove organic matter from NaOH-Na_2_CO_3_ extract, concentrated H_2_SO_4_ was added to precipitate organic matter before P measurement^71,72^.

### Solution Phosphorus-31 Nuclear Magnetic Resonance Spectroscopy

Three grams of the 1989 reference soil and the composite 2015 NK and NPK soils were extracted with 30 mL of 0.25 M NaOH plus 0.05 M Na_2_EDTA extractant for 16 h^77^. After extraction, the samples were centrifuged, and supernatants were frozen and then lyophilized. Lyophilized material was redissolved for P-NMR in 0.65 mL each of D_2_O, DI H_2_O and the NaOH-EDTA extracting solution, plus 0.4 mL of 10 M NaOH. These samples were allowed to stand for 10 min with occasional vortexing and then were centrifuged for 20 min at approximate 1300 *g*, and finally decanted into 10-mm NMR tubes for NMR analysis. Solution P-NMR spectra were obtained at the Saskatchewan Structural Sciences Centre (University of Saskatchewan, Saskatoon, Canada) using a Bruker Avance 500-MHz spectrometer. The NMR parameters were: 90° pulse, 0.68-s acquisition time, 5-s pulse delay, 12-Hz spinning, 20 °C, 2200 to 2900 scans (3–4 h), and no proton decoupling.

To facilitate peak identification, a spiking experiment was conducted using *myo*-inositol hexaphosphate (*myo*-IHP), choline phosphate, glucose-1-phosphate, α- and β-glycerophosphate (all purchased from Sigma-Aldrich). The chemical shift of the orthophosphate peak was standardized to 6 ppm. Signals were assigned to individual P compounds or functional groups based on the spiking experiment and other publications^31,56^. Signal areas were calculated by integration using NUTS software (Acorn NMR). Spectra were plotted with a line broadening of 7 Hz for the overall spectrum and 3 Hz to preserve fine resolution in the orthophosphate monoester region.

### Synchrotron-based techniques

Synchrotron-based bulk-XANES and microprobe experiments were conducted at the Soft X-ray Micro characterization Beamline (SXRMB) equipped with Si(111) double-crystal monochromators at Canadian Light Source (CLS), Saskatoon, Canada. The operating condition of the storage ring in CLS was 2.9 GeV with a maximum current of 250 mA. Detailed information on sample and P standard preparation and data collection for bulk-XANES was described previously^13^. Additional two standards, including IHP adsorbed on boehmite and ferrihydrite, were prepared at pH 5.0 using batch experiment following the procedures reported by Prietzel *et al*.^78^. Briefly, the 1989-reference soil and composite soil samples collected in 2015 were finely ground and mounted as homogeneous thin films on a P-free carbon tape. The soil spectra were collected in partial fluorescence yield (PFY) mode using a four-element Si-drift. Standard spectrum of HAP was collected to calibrate the absolute energy scale of all sample spectra to 2151.4 eV (*E*_0_), and then all the sample spectra were well aligned to our published standard spectra^13^ for the subsequent LCF analysis.

After bulk-XANES measurements, the microprobe at this beamline was applied to collect the µ-XRF images of the two soils with NK and NPK fertilization sampled in 2015. Detailed information on the microprobe setting and applications were recently reported^35^. In brief, the two soil samples attached on carbon tapes were transferred to the chamber for microprobe measurements, and then were scanned at a step size of 50 µm × 50 µm (i.e. coarse scan) using a focused X-ray beam. Fluorescence signals of multiple elements including Fe, Ca, Al, and P were recorded during each image scan using a Bruker detector. After coarse scan, two or three sample regions with P hot spots were selected to scan at a step size of 10 µm × 10 µm (i.e. fine scan). At least two separate µ-XRF maps were collected, but only one representative map was selected in this paper. µ-XANES spectra were collected for at least two P hot spots for each fine scan image of the two soil samples in the PFY mode. Radiation damage during XANES experiment was excluded by a good reproducibility of the repeated measurements on the same spot and repeated scans over different spots for each sample.

All XANES spectra were background corrected and normalized by ATHENA^79^. For bulk-XANES spectra, LCF of soil spectra was performed across the spectral energy region from 2140 to 2185 eV using all possible binary, ternary and quandary combinations of all collected standard spectra with fixed *E*_0_. Weights of all P standards used were forced to sum to 1. The goodness-of-fit was judged by the chi-squared values and R values, and P standards yielding the best fit were considered as the most probable P species in the investigated soil samples. For the microprobe data, SMAK software was used to generate P, Ca, Fe and Al images of the soil samples.

## Electronic supplementary material

supplementary information
