# Supplementary material for: Molecular speciation and transformation of soil legacy phosphorus with and without long-term phosphorus fertilization: Insights from bulk and microprobe spectroscopy
Source: Sci Rep. 2017 Nov 10;7:15354. doi: 10.1038/s41598-017-13498-7 (PMC5681624; doi:10.1038/s41598-017-13498-7)
Supplement: Supplementary file 1 — supplementary information [file 41598_2017_13498_MOESM1_ESM.pdf]

Supplementary Information

Molecular speciation and transformation of soil legacy phosphorus with and without long-term phosphorus fertilization: Insights from bulk and microprobe spectroscopy

Jin Liu[1], Jianjun Yang[2], Barbara J. Cade-Menun[3], Yongfeng Hu[4], Jumei Li[1], Chang Peng[5], Yibing Ma[1*]

[1] Institute of Agricultural Resources and Regional Planning, Chinese Academy of Agricultural Sciences, Beijing, 100081, China;

[2] Institute of Environment and Sustainable Development in Agriculture, Chinese Academy of Agricultural Sciences, Beijing, 100081, China;

[3] Agriculture and Agri-Food Canada, Swift Current Research and Development Centre, Box 1030, Swift Current, SK S9H 3X2 Canada;

[4] Canadian Light Source, University of Saskatchewan, Saskatoon, SK S7N 2V3 Canada;

[5] Agriculture Environment and Resources Center, Jilin Academy of Agricultural Sciences, Jilin 130033, China.

*Corresponding author (mayibing@caas.cn).

Figures: 4

Tables: 2

TABLE S1. Selected chemical properties of the studied soil samples including the reference soil collected in 1989 and those collected in 2015 from long-term plots with (NPK) and without (NK) P fertilization for 27 years (means ± standard errors, n=3)[a].

Treatment	pH	Total C	Total N	Total P	Organic P		Olsen-P
	(2.5 water/soil)	g kg^{-1}	g kg^{-1}	mg kg^{-1}	mg kg^{-1}	%	mg kg^{-1}
1989-Reference	7.6±0 a	13.9±0.5 a	1.3±0 a	490.0±10.0 b	200.1±1.9 b	40.8±0.4 b	12.0±0.7 b
2015-NK	5.7±0.3 b	15.8±0.9 a	1.4±0.1 a	440.5±10.5 c	251.1±6.1 a	57.0±2.7 a	4.6±0.3 c
2015-NPK	5.8±0.2 b	15.8±0.3 a	1.5±0 a	654.6±14.0 a	235.7± 8.8 a	36.0±0.7 b	44.0±5.0 a

[a]Values in each column followed by the same letters are not significantly different ($P < 0.05$).

TABLE S2. Phosphorus forms[a] in the NaOH-EDTA extractions as determined by integration of P-NMR signals from the studied soils.

Treatment[b]	Ortho	Pyro	Poly	Phon	myoIHP	ScyIHP	neoIHP	ChiroIHP1	ChiroIHP2	α-glyc	β-glyc
Phosphorus proportions (%)											
1989-Reference	41.6	0.8	2.3	4.6	8.2	1.4	2.1	2.7	5.5	4.1	2.1
2015-NK	30.2	3.8	0.8	3.0	8.2	2.1	2.7	4.1	7.5	4.8	2.0
2015-NPK	62.4	2.3	0.8	3.1	4.2	0.7	2.1	1.4	4.2	2.8	1.4
Phosphorus concentrations (mg kg^{-1})											
1989-Reference	67.8	1.3	3.7	7.5	13.4	2.3	3.4	4.4	9.0	6.7	3.4
2015-NK	53.2	6.7	1.4	5.3	14.4	3.7	4.8	7.2	13.2	8.4	3.5
2015-NPK	201.6	7.4	2.6	10.0	13.6	2.3	6.8	4.5	13.6	9.0	4.5
Changes of P concentrations in treatments relative to 1989 reference (%)[c]											
Without P fertilizer	-22	+413	-62	-30	+7	+62	+39	+64	+47	+26	+3
With P fertilizer	+197	+470	-31	+34	0	-1	+98	+3	+51	+35	+32

Treatment[b]	Nucl	Pchol	g1P	g6P	Mono1	Mono2	Mono3	DNA	OthDi1	OthDi2
Phosphorus proportions (%)										
1989-Reference	2.7	0.7	0	1.4	0.7	12.3	1.4	3.1	1.5	0.8
2015-NK	4.1	1.4	0.7	2.1	0.7	15.8	0.7	3.0	1.5	0.8
2015-NPK	2.1	0.7	0	0.7	0.7	5.7	0.7	1.6	1.6	0.8
Phosphorus concentrations (mg kg^{-1})										
1989-Reference	4.4	1.1	0	2.3	1.1	20.0	2.3	5.1	2.4	1.3
2015-NK	7.2	2.5	1.2	3.7	1.2	27.8	1.2	5.3	2.6	1.4
2015-NPK	6.8	2.3	0	2.3	2.3	18.4	2.3	5.2	5.2	2.6
Changes of P concentrations in treatments relative to 1989 reference (%)[c]										
Without P fertilizer	+64	+116	N/A[d]	+62	+8	+39	-46	+4	+8	+9
With P fertilizer	+54	+98	N/A[d]	-1	+98	-8	-1	+2	+111	+98

[a] orthophosphate (Orth), pyrophosphate (Pyro), polyphosphate (Poly),phosphonates (Phon), *myo*-inositol hexakisphosphate (myoIHP), *scyllo*-inositol hexakisphosphate (ScyIHP), *neo*-inositol hexakisphosphate (neoIHP),*chiro*-inositol hexakisphosphate 4e/2a (ChiroIHP1),*chiro*-inositol hexakisphosphate 2e/4a (ChiroIHP2),a and b glycerophosphate(a-glyc and b-glyc, respectively), mononucleotides (Nucl), choline phosphate(Pchol), glucose 1-phosphate (g1P), glucose 6-phosphate (g6P), orthophosphate monoesters, regions 1, 2, and3 (Mono1, Mono2, and Mono3, respectively), deoxyribonucleic acid (DNA), and orthophosphate diesters, regions 1 and 2 (OthDi1and OthDi2, respectively). [b] 1989-Reference, the soil sampled before the establishment of the experiment as a reference, 2015-NK and NPK, the soil sampled in 2015 from the long-term experiment with 27-year no and continuous P fertilization, respectively. [c] The ratio of differences in concentrations of each P species between the 2015 samples with and without P and the 1989 reference soil, expressed in percentage. [d] not applicable since the denominator was zero.

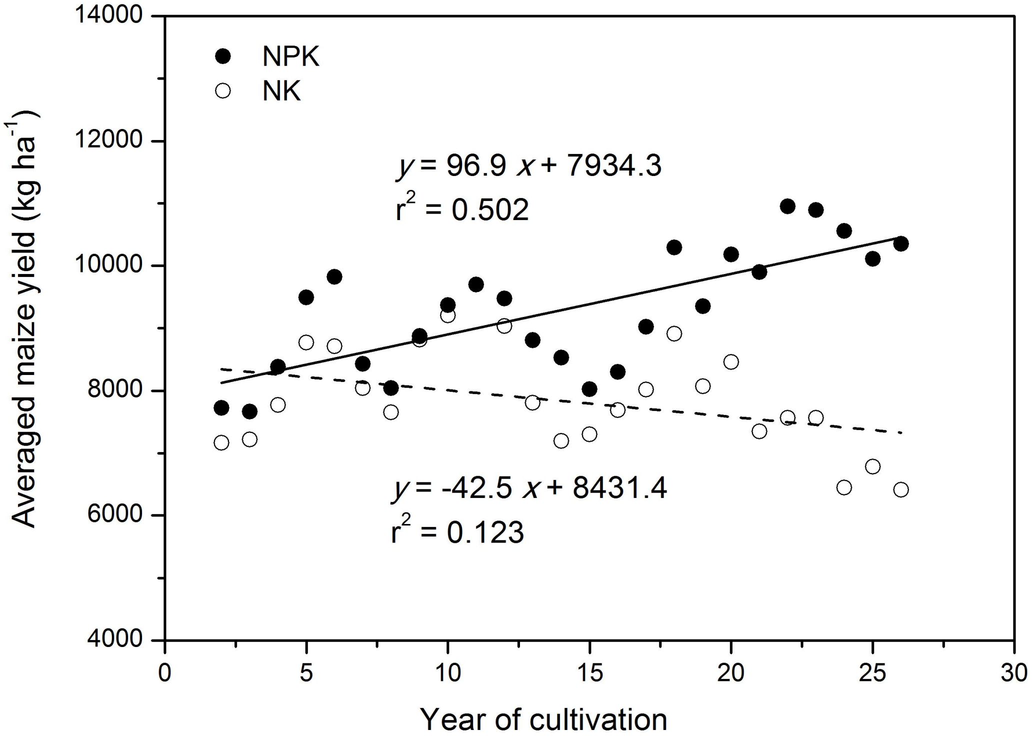

Fig.S1. Three-year averages of maize yield of the long-term experiment with (NPK, solid circles) and without (NK, open circles) P fertilization. Fitted curves for NPK (solid line) and NK (dash line) treatment: y=a*x*+b.

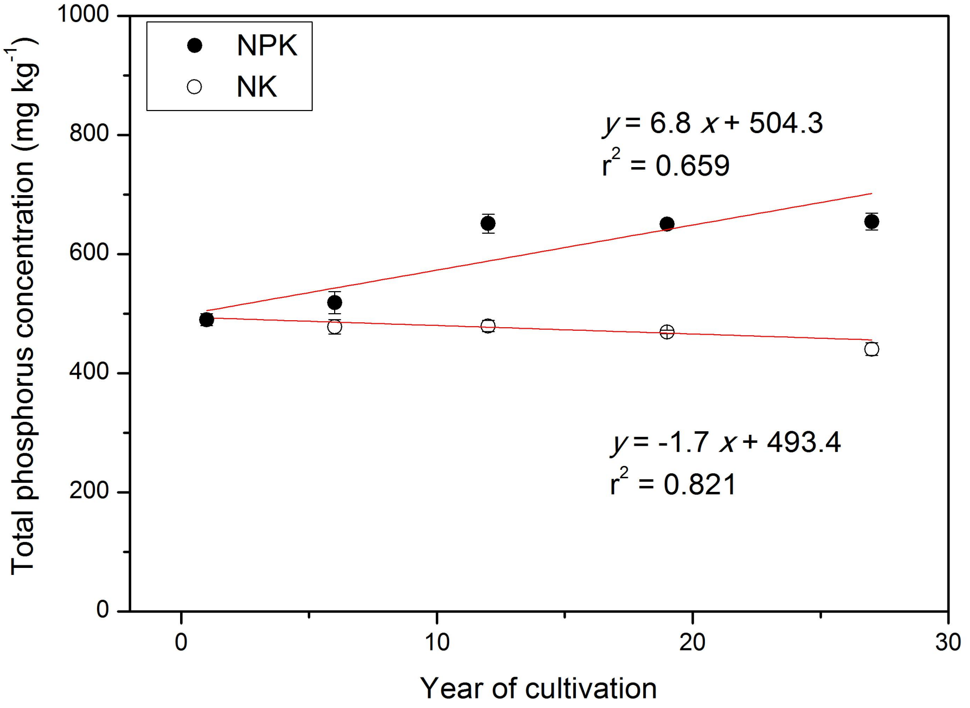

Fig.S2. Change in total phosphorus concentration of the studied soils from the long-term experiment with (NPK, solid circles) and without (NK, open circles) P fertilization. Fitted curves for each treatment: y=a*x*+b.

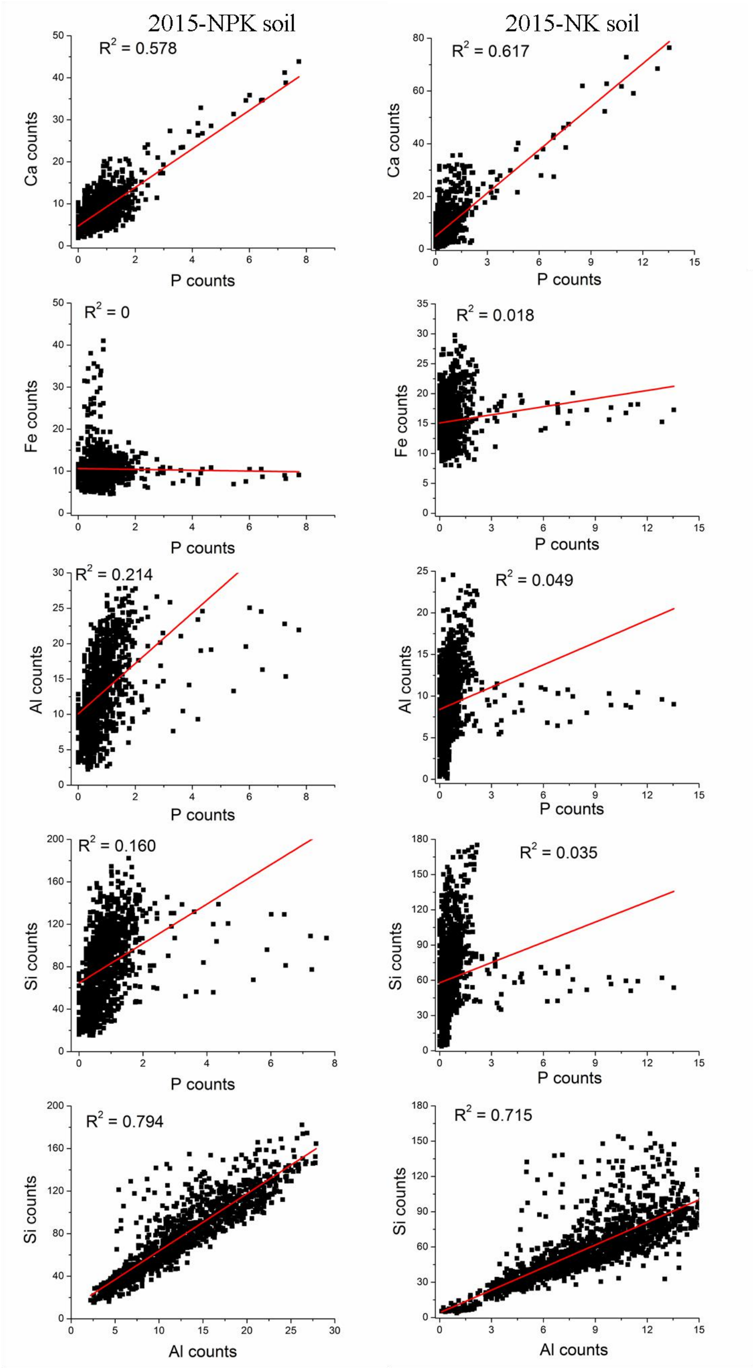

Fig.S3. Correlations between the fluorescence signals of P vs. its potential bonding elements (Ca, Fe, Al, Si) and Al vs. Si. Each point represents a pixel in Fig. 3.

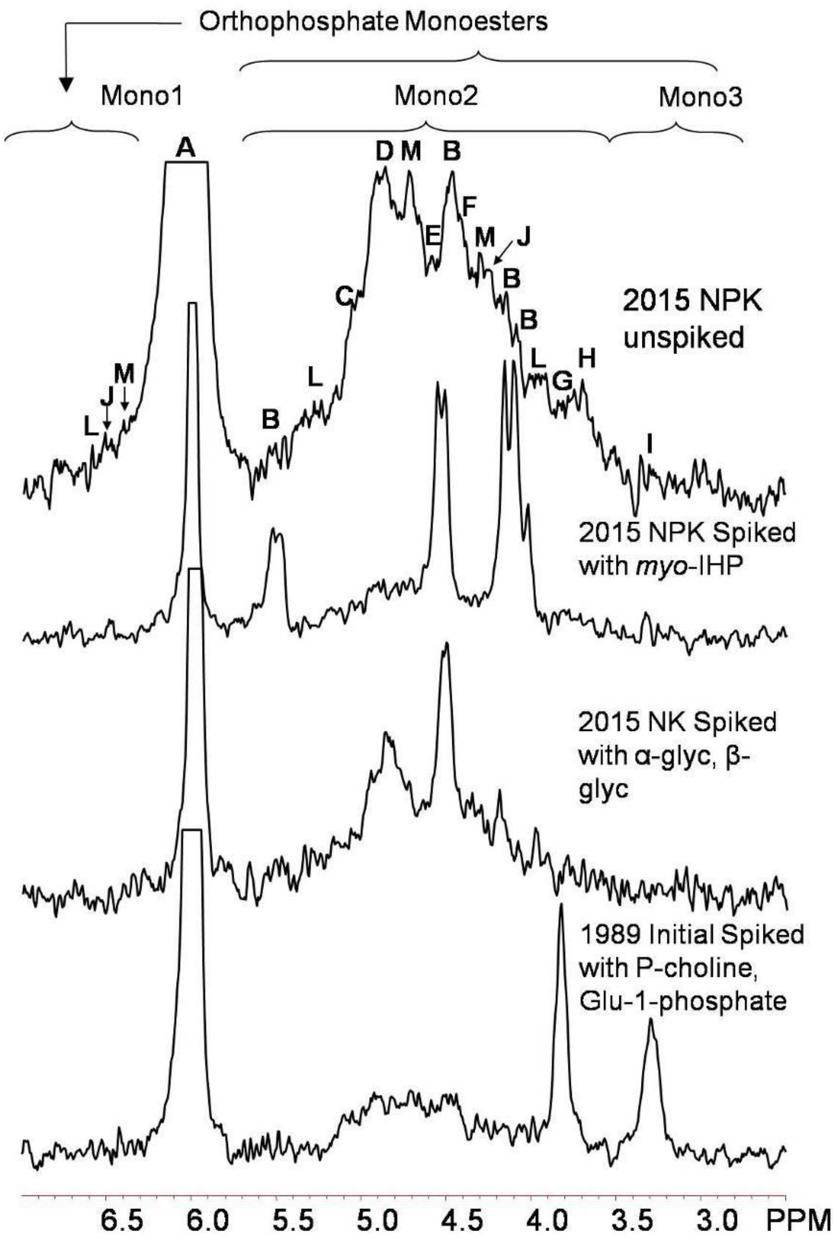

Fig. S4. P-NMR spectra showing the assignment of peaks in the orthophosphate monoester region, including spiking experiments. A, orthophosphate; B, *myo*-inositol hexakisphosphate (IHP or phytate); C, glucose 6-phosphate; D, α-glycerophosphate; E, β-glycerophosphate (β-glyc); F, mononucleotides; G, choline phosphate (P- choline); H, *scyllo*-inositol hexakisphosphate; I: glucose 1-phosphate; J: *neo*-IHP 4equatorial/2axial configuration; K: *neo*-IHP 2equatorial/4axial configuration; L: D-*chiro*-IHP 4equatorial/2axial configuration; M: D-*chiro*-IHP 2equatorial/4axial configuration; Mono1, Mono2, and Mono3: Other unidentified orthophosphate monoesters in regions 1, 2, and 3.
